# Pulmonary artery catheter entrapment in cardiac surgery

**DOI:** 10.4103/1658-354X.65125

**Published:** 2010

**Authors:** Raed Alsatli

**Affiliations:** *Assistant Professor, Department of Cardiac Science, Consultant Cardiac Anesthetist, College of Medicine King Saud University, Riyadh, KSA*

**Keywords:** *Cardiac surgery*, *PAC entrapment*, *pulmonary artery catheter*

## Abstract

A pulmonary artery catheter (PAC) is an important tool in the preoperative cardiac management, and it provides measurements which helps in the patient management During open heart surgery the catheter tends to rest against the anterior lateral wall of the right atrium where the catheter may be caught by a suture in the cannulation for cardiopulmonary bypass. We describe a very rare complication which is inadvertent surgical suturing of the PAC to the inferior vena cava that necessitated reopening the chest, cutting, the suture and removing the catheter.

## INTRODUCTION

A pulmonary artery catheter (PAC) is an important tool in the preoperative cardiac management, and it provides the following measurements, central venous pressure, pulmonary artery pressure, cardiac output, systemic vascular resistance, pulmonary vascular resistance, wedge pressure, and mixed venous oxygen saturation. The reported complications of PAC are bleeding, infection, fragmentation, and knotting.

Entrapment of the PAC to an intra cardiac structure is a very rare but serious complication. During open heart surgery the catheter tends to rest against the anterior lateral wall of the right atrium where the catheter may be caught by a suture in the cannulation for cardiopulmonary bypass (CPB).[1]

We describe a very rare complication which is inadvertent surgical suturing of the PAC to the inferior vena cava that necessitated reopening the chest, cutting the suture and removing the catheter.

## CASE REPORT

A 53-year-old male patient was presented who underwent mitral valve replacement surgery. He presented with a history of rheumatic mitral valve disease, hypertension, shortness of breath, and pulmonary edema. Echocardiography showed in addition to normal left ventricular function, severe mitral regurgitation moderate aortic regurgitation and mild right ventricular dysfunction. Because of positive troponine, cardiac catheterization was performed and showed normal coronaries. Vital signs were within the normal range. The patient was receiving the following medications: bisoprolol, furosemide, lipitor, and lisinopril. All laboratory values were within the normal range.

**Figure 1:**
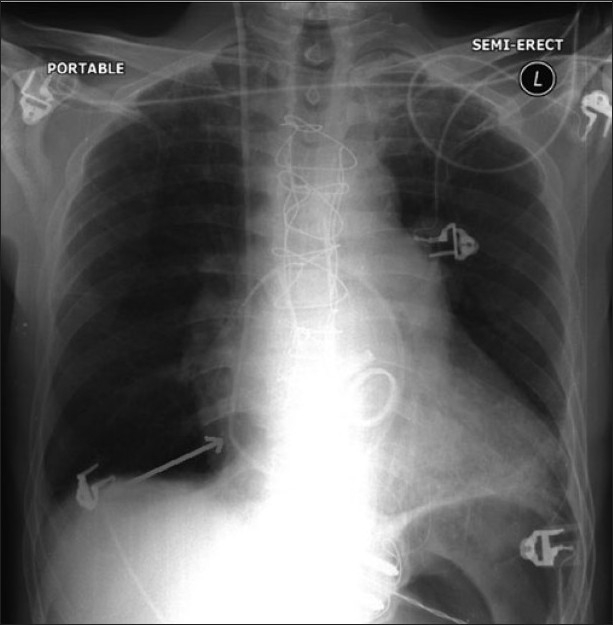
Chest radiograph on the first postoperative day. Arrow shows angulation of the PAC

The patient was then scheduled for double valve replacement. In the operating room, the radial artery was cannualated, and induction was performed with midazolam 1.2 mg/kg, sufentanil 2 µg/kg, and rocuronium 1 mg/kg. The trachea was intubated and lung ventilated. The PAC was inserted smoothly in the right internal jugular vein. Continuous infusion of midazolam 30 mcg/kg/h, rocuronium 0.5 mg/kg/h, and sufentanil 0.3 mcg/kg/h was started. Double valve replacement was performed through median sternotomy. Surgery was uneventful and the patient was shifted to intensive care unit where he was fully stable and the trachea was extubated few hours later. The patient was stable on the first postoperative day, and it was planned to remove the PAC but there was resistance on pulling the PAC. Chest radiograph did not show abnormal finding except an angulation in the course of the PAC in the lower part of the heart shadow, which gave a possible diagnosis of PAC suture entrapment [Figure 1]. The patient was uncooperative to perform trans-esophageal echocardiography (TEE). Based on the clinical assessment and chest radiograph, it was decided to remove the entrapped PAC surgically. The patient was transferred to operating room again to remove the PAC.

**Figure 2:**
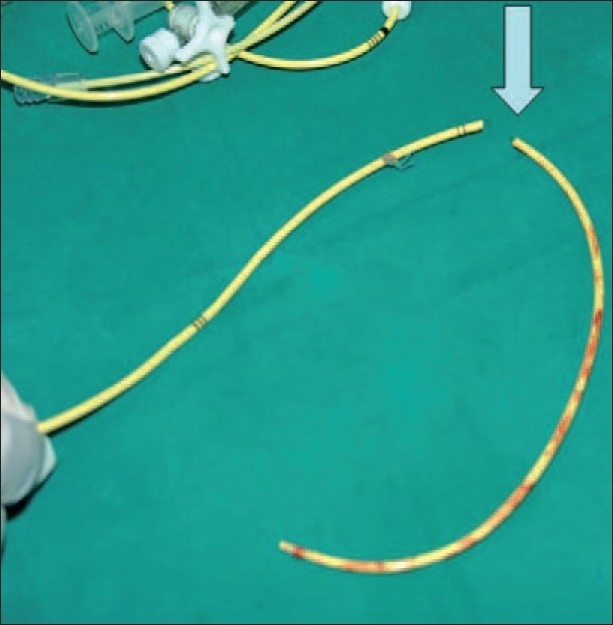
PAC after removal (it was cut by the surgeon at the suture site removal)

After opening the chest PAC pulling showed that it was fixed to a suture in the inferior vena cava purse suture. Very cautiously with the cardiopulmonary pump ready, the surgeon entered his finger inside the right atrium and managed successfully to free the catheter, which was then pulled out completely [Figure 2].

The patient was stable and the trachea extubated post operatively, and he was transferred to the ward on the next day.

## DISCUSSION

A PAC is an important tool, which can be useful in the management of patients undergoing cardiac surgery and diagnosis of hemodynamic instability.

Indications for using it include cardio-surgical patients, non cardiac surgery patients with severe left ventricular dysfunction or sever pulmonary hypertension, in the setting of intensive care medicine: septic shock, cardiogenic shock, pulmonary edema, and sever toxemia of pregnancy. In a review of 6245 patients undergoing pulmonary artery catheterization, Shah *et al*, reported extremely low rates of morbidity resulting from the PAC itself.[2] Complications of the PAC include tachyarrhythmia, complete heart block, infections, catheter knotting, and pulmonary artery rupture.

PAC entrapment to intracardiac structures during open heart surgery has been described in the literature, most of the catheter entrapment happened in the right atrium. In this case, the PAC was sutured surgically to the wall of the inferior vena cava while doing the purse suture for inserting the inferior vena cava cannula prior to CPB and necessitated re-exploration of the chest to remove it.

This is a rare situation. There are other case reports of suture entrapment to purse string of superior vena cava, pulmonary artery,[3] retrograde cardioplegia cannula in the right atrium, and to the venting cannula.[4,5] It has to be stressed here that trials to remove the PAC was done very gently, as forceful traction on the PAC may lead to rupture of the area where the PAC is fixed, leading to fatal bleeding. Possible causes of resistance while removing the PAC are catheter knotting, catheter deformation, and suture entrapment.[6,7] On pulling the entrapped PAC, pulsatile motion can be felt.[8] Acute angulation of the PAC on chest radiograph suggests suture entrapment.[9] Fluoroscopy can be helpful in diagnosing the reason for entrapment.[10] TEE is an important tool, which shows deformity of heart contour wall when traction is applied on the PAC.[11,12] In this case PAC entrapment was based on the angulation of PAC which was obvious on the plain chest radiograph. TEE is an important tool for the diagnosis, but could not be done because the patient was uncooperative. As the gentle trial failed to move the PAC it was decided to remove it surgically. If the PAC has not been stitched, non-surgical procedures to remove the PAC include stone retriever basket, and if the catheter is tethered by a simple stitch around it, simple slipping through the stitch might be successful. In this case although the PAC was pulled about 5 cm at the end of CPB there was bleeding from the site of the purse string after removing the CPB cannula from the inferior vena cava after weaning from CPB; therefore, the surgeon did extra sutures to stop the bleeding from this place, probably at this stage, the PAC was sutured.[9]

In conclusion, we do recommend pulling the PAC 5 to 10 cm at termination of CPB to ensure that it is free. Also trial to pull the PAC has to be very gentle, and under no circumstances force is applied. Chest X-ray can be helpful in the diagnosis of PAC entrapment due to suture fixation. Angulation of the catheter is an important diagnostic sign. Finally, if gentle pulling of the catheter fails, then re-exploration is recommended for catheter removal.
